# Clinical and functional significance of CHK1-S, an alternatively spliced isoform of the CHK1 gene, in hepatocellular carcinoma

**DOI:** 10.7150/jca.39443

**Published:** 2020-01-17

**Authors:** Guanghui Hu, Shuren Wang, Yu Wang, Yang Gao, Hongxia Zhu, Mei Liu, Ningzhi Xu, Liming Wang

**Affiliations:** 1 Laboratory of Cell and Molecular Biology & State Key Laboratory of Molecular Oncology, National Cancer Center/National Clinical Research Center for Cancer/Cancer Hospital, Chinese Academy of Medical Sciences and Peking Union Medical College, Beijing, China.; 2 Department of Hepatobiliary Surgery, National Cancer Center/National Clinical Research Center for Cancer/Cancer Hospital, Chinese Academy of Medical Sciences and Peking Union Medical College.

**Keywords:** CHK1, CHK1-S, HCC, SRPK1, RFS

## Abstract

Alternative splicing plays critical roles in many disease processes and splicing dysregulation is a hallmark of cancer. The different splicing isoforms may have significantly different effects on the malignant progression of cancer. Checkpoint kinase 1 (CHK1) is a serine/threonine kinase and regulates DNA damage response. In this study, we measured the expression of an alternative CHK1 transcript (CHK1-S, excluded exon 3) in hepatocellular carcinoma (HCC) tissues. Our results showed that CHK1-S was significantly upregulated in HCC tissues compared with paired adjacent noncancerous hepatic tissues. The levels of full-length CHK1(CHK1-L), CHK1-S and the ratio of CHK1-S/L in tumor tissue were associated with relapse free survival (RFS) of postoperative HCC patients, respectively, but not the levels of CHK1-L, CHK1-S and the ratio of CHK1-S/L in adjacent normal tissue. To further demonstrate the role of CHK1-S in HCC, CCK-8 assays, EdU incorporation assays and colony formation assays were used. The results showed that overexpression of CHK1-S significantly accelerated HCC cell proliferation, compared with CHK1-L. In addition, we found that serine-arginine protein kinase 1 (SRPK1), as an upstream regulator kinase of splicing factor, could upregulate the expression of CHK1-S and its expression level was significantly higher in HCC tumors than the paired normal tissues and was associated with the levels of CHK1-S (P=0.016). In conclusion, our study demonstrated that CHK1-S, acts as an oncogene, which was upregulated and associated with RFS in HCC patients. SRPK1 may mediate its mRNA splicing in HCC. All these data indicated that the expression of CHK1-S would have potential prognostic values and splicing kinase SRPK1 might be developed as therapeutic target in HCC.

## Introduction

Hepatocellular carcinoma (HCC) is one of the most prevalent primary liver cancer and the major cause of cancer death in China 1, 2. Though many curative therapies have been developed, the overall response to these therapies leaves much to be desired and the long-term prognosis of HCC patients remains poor owing to its high recurrence rates 3. Deciphering the molecular mechanisms of HCC initiation and progression may give rise to improved diagnostic and therapeutic strategies. Therefore, it is urgently needed to identify reliable early diagnostic biomarkers and effective treatment options.

Alternative splicing generates different mature mRNAs through the inclusion or exclusion of specific exons to increase transcriptome and proteome diversity 4. Alternative splicing has critical roles in normal development, while its dysregulation is commonly observed in numerous diseases, including cancer 5-7. Cancer-specific mRNA transcripts, regulated by trans-acting splicing factors, may result in either loss-of-function of tumor suppressor genes or activation of oncogenes, and promote cell proliferation or survival, eventually leading to tumor invasion and metastasis 8. Induced by amphiregulin, p73 is alternatively spliced into its oncogenic isoform, ΔEx2p73, during liver tumorigenesis 9. Two Numb splice isoforms, which differ in inclusion (long, PRRL) or exclusion (short, PRRS) of exon 12, have opposite effects on HCC cell proliferation 10. The ^△2-4^Merlin splice variant that lacks exons 2, 3 and 4 disrupts the normal function of Merlin and promotes hepatocellular carcinoma metastasis 11.

Checkpoint kinase 1 (CHK1) is a serine/threonine protein kinase originally identified as a regulator that controls the G2/M phase transition in response to DNA damage in yeast 12. CHK1 plays a critical role in sensing the initial single strand DNA breaks to activate DNA damage response 13. Activated CHK1 phosphorylates its downstream targets to bring about cell cycle arrest, activate DNA repair pathways, and induce apoptosis when DNA damage is severe 14-17. Recently, it has been reported that CHK1 is overexpressed in HCC tissues compared with adjacent non-tumour tissue 18. In addition, previous study has reported that CHK1-S, an alternative splice variant of CHK1, interacts with CHK1 and acts as an endogenous inhibitor of CHK1 19. The study has also showed that CHK1-S expression correlates with the degree of malignancy in ovarian and testicular tumors 19. However, there has hitherto been a lack of information regarding the status of CHK1-S in HCC.

In this study, we demonstrated that CHK1-S was upregulated in HCC tissues. High CHK1-S (which lacks exon 3) and CHK1-L (full-length CHK1) expression were significantly associated with worse relapse free survival (RFS) of postoperative HCC patients, besides, high ratio of CHK1-S/L in HCC tissues correlated with reduced RFS. CHK1-S functioned as an oncogene in promoting tumor growth. SRPK1 may be the splicing modulator that regulates the alternative splicing of CHK1 in HCC.

## 2. Materials and methods

### 2.1 Tissue samples

HCC tissues and the paired adjacent noncancerous hepatic tissues were obtained from 54 patients who underwent radical resections in the Cancer Hospital (Chinese Academy of Medical Sciences & Peking Union Medical College) between May 2012 and April 2015. In this retrospective study, the process does not interfere any diagnosis and treatment, the results will not contain any identifiable patient's information and be published as statistically analyzed data. Moreover, some of the included patients have died and it is impossible to obtain informed consent. Thus, the Ethics Committee approved the study and permitted to waive the informed consent of all the patients in this study (3332018193). The clinical characteristics of the patients were shown in Table 1.

### 2.2 Plasmids

The cDNA obtained from 293T cells was used as template to amplify genes of interest in the course of subsequent clonings. Human CHK1 gene (GenBank: NM_001114122.2 or NM_001330428.1) encoding full length or excluded exon 3 were amplified by PCR and subcloned into pcDNA3.1-Myc/His B(-) vector, respectively. All the primers used for cloning were enlisted in Table 2. Human SRPK1 plasmids (pReceiver-M35-C-Flag) were bought from Fulen Gene (Guangzhou, China).

### 2.3 Cell cultures

The human HCC cell lines HepG2 and QSG-7701 were obtained from Cell Resource Center (Beijing, China). The cells were grown in Dulbecco's modified Eagle's medium (DMEM) (Shengwuxigong Company, Beijing ,China) containing 10% fetal bovine serum (Invitrogen) and maintained at 37℃ in an atmosphere containing 5% CO_2_. For construction of stably overexpressed cells, pLVX CHK1-S, pLVX CHK1-L were transfected into HepG2 cells and selected with puromycin (2 mg/ml) for one week.

### 2.4 Real-time PCR

Total RNAs were extracted using the Trizol reagent (Invitrogen) following the manufacturer's instruction. cDNA was synthesized using PrimeScript™ RT reagent Kit (Takara, RR047A). PCR reactions were carried out on a StepOnePlus™ Real-time PCR System (Applied Biosystems, Carlsbad, CA, USA) in 20 μl reaction volume. The primers used were enlisted in Table 2. TaqMan-based real-time PCR was conducted for detecting CHK1-L and CHK1-S expression. SYBR-based real-time PCR was conducted for detecting SRPK1 expression.

### 2.5 Antibodies and Immunoblot Analysis

Extraction of proteins from cultured cells or tissues using RIPA buffer was followed by immunoblot analyses. The following antibodies were used for initial immunoblot analysis. CHK1 (1:1000, 2360, Cell Signaling Technology), SRPK1 (1:1000, 14073-1-AP, Proteintech), beta-Actin (1:1000, 60008-1-Ig, Proteintech). After extensively washed with TBS-T buffer, the membranes were then incubated with secondary antibodies (ZSGB-BIO, China) for 1 h at room temperature. Protein expression levels were detected using enhanced chemiluminescence (Engreen, China) according to the manufacturer's instructions.

### 2.6 Cell proliferation assays

Cell proliferation was assessed using Cell Counting Kit-8 and Ethynyl deoxyuridine (EdU) incorporation assays. For cell viability, a total of 4000 HepG2 cells were plated in 96-well plates and measured using the CCK-8 (Dojindo Laboratories, Japan) following the manufacturer's instructions. The cell proliferation curves were plotted using the luminescence at each time point. EdU incorporation assays were performed with an EdU kit (Beyotime Company, China) following the manufacturer's instructions. The images were obtained by Confocal Microscope (Leica, Germany).

### 2.7 Colony formation assays

One thousand HepG2 cells were seeded per well in 6-well plates and incubated for 10 days with normal DMEM medium. The colonies were fixed by 0.4% PFA and stained with 0.5% crystal violet.

### 2.8 Microarray data analysis

The Affymetrix GeneChip Human Transcriptome Array (HTA2.0) were used to identify differentially expressed genes of paired tumor and non-tumor tissues from five HCC patients with recurrence within 1 year after primary resection. The RNAs were isolated using Trizol reagent. Total RNA was converted to cDNA and then hybridized to Affymetrix HTA 2.0 arrays according to the manufacturer's instructions. The raw data of microarray was pretreated by Expression Console software. We performed a Student's paired t-test to compare gene intensities in the different tissue samples. Genes that had fold changes of ≥ 2.0 and uncorrected p-value <0.05 were considered significantly regulated.

### 2.9 Statistical analysis

For comparisons, Wilcoxon signed-rank test, Student's t-test, and Mann-Whitney test were performed as indicated. *P*-values were obtained with the SPSS 18.0 software (SPSS, USA). *P*-values <0.05 were defined as statistically significant.

## 3. Results

### 3.1 Clinical significance of splicing isoform CHK1-S in hepatocellular carcinoma

**Figure 1:**
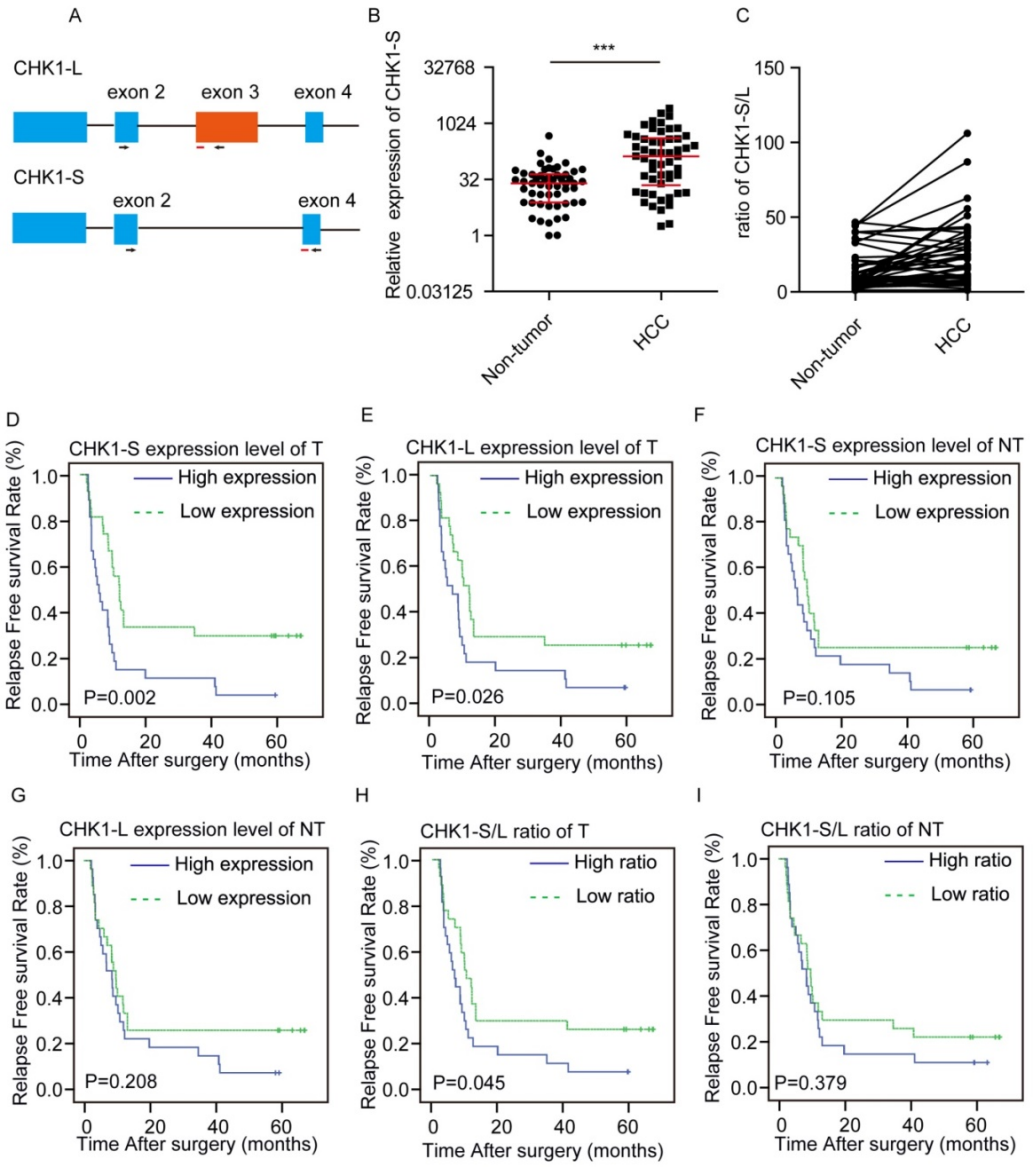
** Clinical significance of CHK1-S in HCC.** (A) Schematic showing alternative splicing of CHK1. Arrowheads and red lines respectively represent the primers and the probes used for splicing assays with quantitative PCR. (B) CHK1-S expression in human HCC tissues and paired adjacent noncancerous hepatic tissues was examined by quantitative PCR. n = 54, *P* = 0.007 by Mann-Whitney test. (C)The ratio of CHK1-S/L (CHK1-S/CHK1-L) in 54 paired human HCC tissues and adjacent noncancerous hepatic tissues. The mRNA expression of CHK1-S and CHK1-L were examined by real-time qPCR. A paired two-tailed Student's t-test was used. *P*= 0.0001. (D-I) Kaplan-Meier curves depicting relapse free survival (RFS). Expression values or ratios were determined for all HCC samples indicated within the cohort. Samples were stratified based on median expression values or ratios. Plots compare the RFS for CHK1-S mRNA level in tumor tissues (D), CHK1-L mRNA level in tumor tissues (E), CHK1-S mRNA level in adjacent non-tumor tissues(NT) (F), CHK1-L mRNA level in adjacent NT tissues (G) CHK1-S/L ratio in tumor tissues (H), CHK1-S/L ratio in adjacent NT tissues (I), respectively. The *P* values were calculated using the log-rank test.

We examined the expression level of CHK1-L (full-length CHK1) and CHK1-S (the shorter isoform of CHK1, which lacks exon 3) in HCC tumors (T) and paired surrounding nontumor tissues (NT) from 54 patients by real-time PCR, respectively (Fig. 1A). We found that CHK1-S mRNA expression was significantly higher in HCC tumors compared with matched non-tumor tissues (Fig. 1B), and CHK1-S/L ratio (CHK1-S/CHK1-L in mRNA level) was higher in HCC than paired non-tumor tissues (Fig. 1C). Above all, CHK1-S, a splicing isoform of CHK1, was upregulated in HCC tissues.

To investigate the correlation between CHK1-S and clinical characteristics, we divided the 54 patients into two groups based on the median value of the expression ratio of CHK1 S/L. As shown in Table 1, the clinic-pathological features of HCC patients, including the patient's age, gender, tumor size, microvascular invasion, differentiation, envelope invasion, satellite nodules, cirrhosis and AFP, have no significant difference between the low and high CHK1-S/L ratio group(*P*>0.05).

In order to further study the relationship between CHK1 isoforms and the prognosis of HCC patients, we made univariate analysis and multivariate analysis. As showed in Table 3, univariate analysis indicated that satellite nodules, microvascular invasion, CHK1-S in tumor tissue, the ratio of CHK1-S/L in tumor tissues and adjacent nontumor tissues were significantly associated with RFS of HCC patients, whereas other features, including age at diagnosis, gender, differentiation, cirrhosis, and envelope invasion, AFP, CHK1-L in tumor or adjacent normal tissue were not. For multivariate analysis, the ratio of CHK1-S/L in tumor tissues and microvascular invasion were significantly associated with RFS of HCC patients.

To further investigate relationship of between CHK1-S or CHK1-L in tumor tissues and the prognosis of HCC patients, we made a survival analysis. Kaplan-Meier analysis revealed that both high CHK1-S and CHK1-L mRNA level in HCC tissues correlated with reduced RFS, but not in non-tumor hepatic tissues (Fig. 1D-G). Furthermore, we explored the relationship of the ratio of CHK1-S/L and the prognosis of HCC patients. As shown in Fig. 1H&I, the group of patients with high ratio of CHK1-S/L in tumor tissues had a poor RFS than the group with low ratio, but not significantly in non-tumor hepatic tissues. Collectively, these data demonstrated that CHK1-S was associated with poor prognosis of HCC patients.

### 3.2 CHK1-S significantly promoted the proliferation of HCC cells

**Figure 2:**
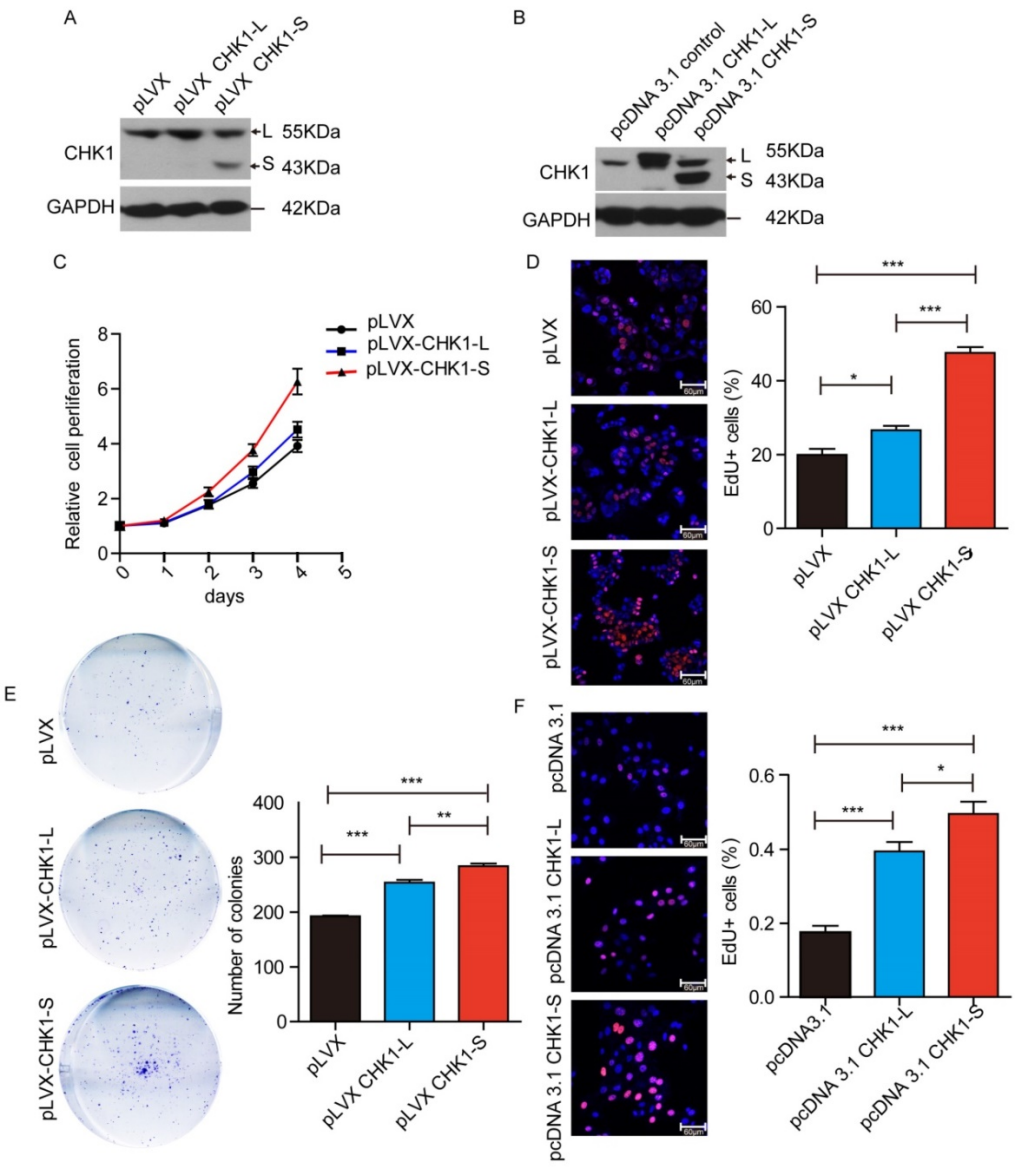
** CHK1-S significantly promoted HCC cell proliferation.** (A&B) Overexpression of CHK1-L and CHK1-S in HepG2 or QSG-7701 cells were examined by western blot. HepG2 cells stably transfected with Lenti-viral empty plasmid pLVX and QSG-7701 cells transient transfected with pcDNA3.1 empty plasmid were used as control, respectively. (C) Cell proliferations were measured using CCK-8 assays in HepG2 cells. (D) Colony formation assays of HepG2 cells stably overexpressing CHK1-S or CHK1-L. Results are shown as the mean ± standard error based on at least three independent experiments. (E&F) Cell proliferations were assessed using EdU immunofluorescence staining in HepG2 (E) or QSG-7701 cells(F). ∗*P* < 0.05, ∗∗*P* < 0.01 by Student's t-test.

To investigate the biological functions of different CHK1 transcripts in HCC, we stably overexpressed CHK1-L or CHK1-S in HepG2 cells by transfecting CHK1-L expression vector (pLVX-CHK1-L) or CHK1-S expression vector (pLVX-CHK1-S) (as showed Fig. 2A). Cell Counting Kit-8 assays showed that ectopic expression of CHK1-S significantly accelerated HepG2 cell proliferation, and while overexpression of CHK1-L slightly promoted the cell proliferation (Fig. 2C). EdU incorporation assays also demonstrated that CHK1-S overexpressed HepG2 cells had more EdU-positive cells than CHK1-L overexpressed cells (Fig. 2D). Furthermore, colony formation assays showed the similar results (Fig. 2E). We also transiently transfected the indicated plasmids into QSG-7701 cells in order to overexpress CHK1-S and CHK1-L, respectively, and the EdU assay showed CHK1-S could accelerate QSG-7701 cell growth than the effect of CHK1-L, as same as the results of HepG2 cells (Fig. 2B&2F). Collectively, these results demonstrated that CHK1-S significantly promoted HCC cell proliferation.

### 3.3 SRPK1 was associated with alternative splicing of CHK1

**Figure 3:**
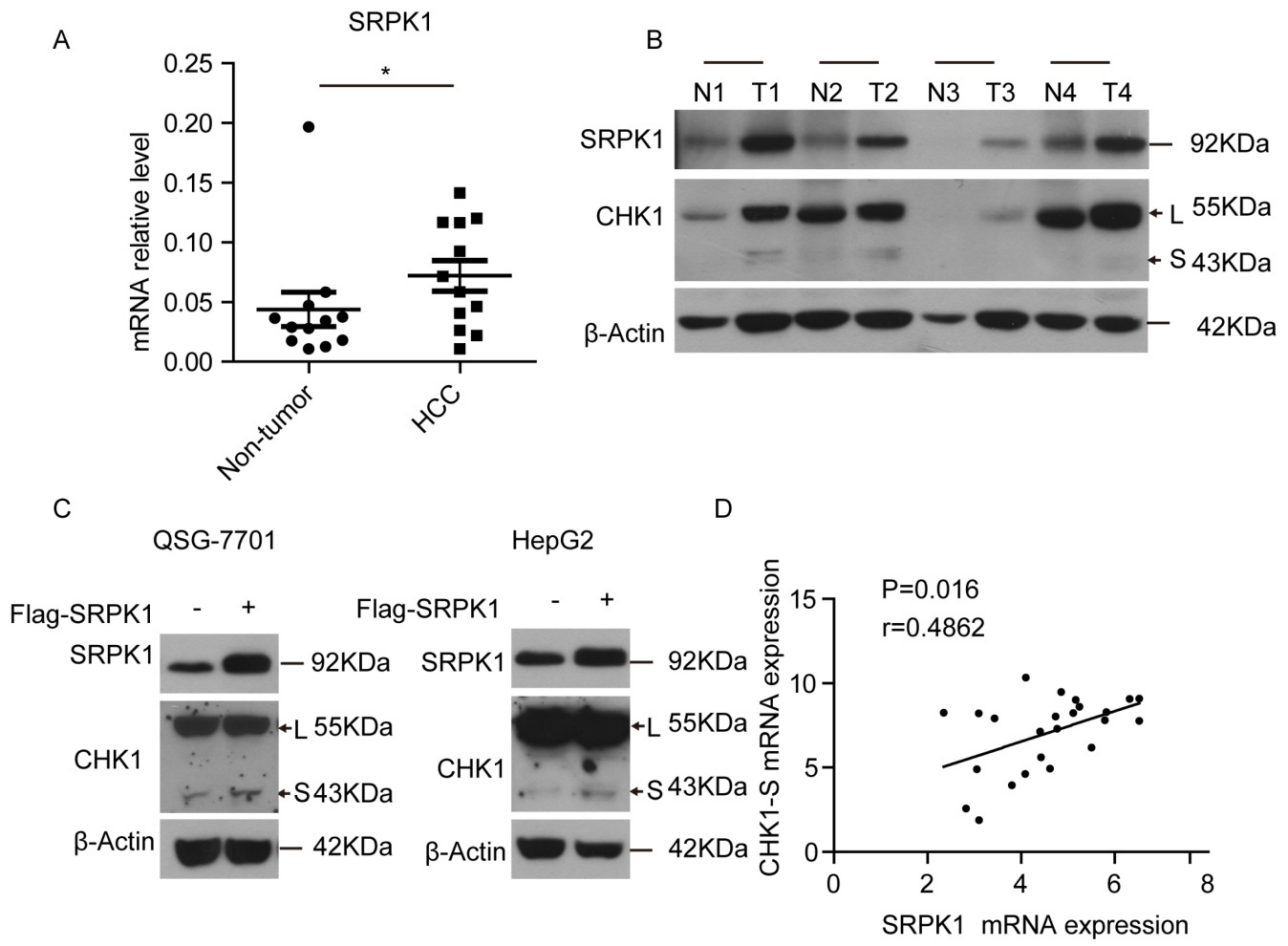
** SRPK1 was associated with alternative splicing of CHK1-S.** (A) SRPK1 mRNA levels in 12 paired HCC and adjacent non-cancerous hepatic tissues. *P* values were acquired by Mann-Whitney test. Data are shown as median with interquartile range. (B) SRPK1 and CHK1 protein levels in 4 paired HCC and adjacent non-cancerous hepatic tissues. (C) Immunoblot analysis of CHK1-S (or CHK1-L) after transient overexpressing SRPK1 in HepG2 and QSG-7701 cells. (D) The correlation between CHK1-S and SRPK1 mRNA level in human HCC tissues (n = 24 samples). *P* < 0.05, r = 0.5807 by Pearson correlation analysis.

To investigate the mechanism underlying CHK1 splicing, we found some RNA binding protein genes (hnRNP A/B, RBM34, SRPK1, etc.) associated with gene alternative splicing were high expressed in HCC tumors through analyzing the microarray data (shown in supplementary table 1). Then we found that SRPK1, as an upstream kinase of splicing factor 20, was significantly higher in HCC tumors compared with matched non-tumor tissues both at the mRNA and protein levels (Fig. 3A&3B). To explore whether the splicing process of CHK1-S is mediated by SRPK1, we transiently overexpressed SRPK1 in HepG2 and QSG-7701 cells, respectively. As shown in Fig. 3C, ectopic expression of SRPK1 significantly increased the protein level of CHK1-S. Besides, we found that SRPK1 mRNA expression levels were significantly correlated with CHK1-S mRNA levels in human HCC tissues (Fig. 3D). These data indicated that SRPK1 may be involved in the alternative splicing of CHK1.

## 4. Discussion

In the present study, we showed that CHK1-S was frequently overexpressed in HCC samples and high expression of CHK1-S and/or CHK1-L, and high ratio of CHK1 S/L in tumor tissue correlated with poor clinical outcome. Compared with CHK1-L, CHK1-S had stronger ability to promote cell proliferation. Furthermore, we found that SRPK1, as an upstream regulator of splicing factor, may be involved in regulating the splicing of CHK1-S.

Many studies showed that the majority function of CHK1 was response to DNA damage, as a cell cycle checkpoint kinase. It induced cell cycle arrest in response to DNA damage mainly by phosphorylating Cdc25 family 21. On the basis of these observations, CHK1 was initially thought to function as a tumor suppressor. However, numerous studies also suggest that CHK1 may actually promote tumor growth at least in some cancers 22-24. Consistent with our results, CHK1 overexpression has been found in many tumors, such as T-cell acute lymphoblastic leukemia, triple-negative breast carcinoma 25, 26. CHK1 may have oncogenic function in HCC, and is mainly detected in the cytoplasm of tumor cells 18. Nuclear CHK1 prevents premature mitotic entry for monitoring of genomic integrity during cell proliferation 27. CHK1 localizes in centrosome and interacts with UNC45A to regulate cancer cell proliferation 28. So, the function of CHK1-S promotes cell proliferation perhaps not only as an activation inhibitor of CHK1-L but also having effect on the subcellular localization of CHK1-L. In our study, the high ratio of CHK1 S/L correlated with poor prognosis of HCC patients, consistent with the previous study that CHK1-S was up-regulated in testicular carcinoma tissues, especially in late-stage tumor samples 19. High expression of CHK1-S could induce high genomic instability and high DNA-repair efficiency for survival in the unperturbed cancer cell, as previous study showed, CHK1 heterozygosity, conditional in the mouse mammary gland, resulted in three distinct haploinsufficient phenotypes that could contribute to tumorigenesis 29. In addition, after comparing the expression of two CHK1 isoform, our data indicated that CHK1-S was just a spoiler, not the major factor for the progression of HCC (Fig. 1C, 3B).

SRPK1 can be activated by Akt and is an important branch to regulate alternative splicing 30. Shuttling splicing factors are phosphorylated in the cytoplasm by SRPK1 and are subsequently transported to the nucleus 20, 31-33. The serine/arginine rich (SR) proteins of splicing factors, such as SRSF1 which is a validated substrate of SRPK1 consequently increasing its nuclear activity and promoting target alternative splicing events, promote pre-mRNA splicing by binding to exonic splicing enhancers (ESEs) in pre-mRNA 34, 35. Previous studies showed that Tra2 contained RS domain (domains enriched in arginine and serine residues) plays a role in regulating CHK1-S splicing 36, 37. Our data showed that SRPK1 was associated with alternative splicing of CHK1 in both cancer cells and tumor tissues. Furthermore, SRPK1 had been reported to associate with hepatocellular carcinoma progression and poor patient survival 38. Therefore, SRPK1 may mediate CHK1-S mRNA splicing through its downstream splicing factor in HCC. Our data also suggested that the SRPK1 protein may provide a novel target to inhibit HCC cell growth.

## 5. Conclusion

In summary, our study demonstrated that CHK1-S was highly expressed in HCC tissues and high CHK1-S expression was correlated with poor prognosis in HCC patients, indicating that CHK1-S could be a prognostic indicator of HCC and reducing CHK1-S expression may be a candidate target for HCC therapy. In addition, SRPK1 may be involved in this process of CHK1 splicing.

## Supplementary Material

Supplementary figures and tables.

## Figures and Tables

**Table 1:** Clinical characteristics of 54 HCC patients according to CHK1-S ratio.

	Low Ratio (n=27)	High Ratio (n=27)	P value
Gender (Male/Female)	22/5	23/4	1.000
Age (years)	55.04±10.00	54.07±13.47	0.767
Tumor size (cm)	4.86±2.00	5.36±2.87	0.461
Microvascular invasion (Present/Absent)	16/11	14/13	0.584
Differentiation (Moderate/Poorly)	21/6	17/10	0.233
Satellite Nodules (Present/Absent)	22/5	23/4	1.000
Envelope invasion (Present/Absent)	23/4	18/9	0.111
Cirrhosis (Present/Absent)	24/3	22/5	0.704
Ln(AFP)	5.27±2.82	4.96±3.31	0.714

**Table 2:** Primer Sequence Information.

	Primer Sequence 5'-3'
pLVX CHK1-S	F-CCGGAATTCATGGAGAAGCCAGACATAGGCA R-GGGGGGCCCTCATGTGGCAGGAAGCCAAATCT
pLVX CHK1-L	F-CCGGAATTCATGGCAGTGCCCTTTGTGG R-GGGGGGCCCTCATGTGGCAGGAAGCCAAATCT
pcDNA 3.1 CHK1-S	F-CCGGAATTCATGGAGAAGCCAGACATAGGCA R-CGGGGTACCGATGTGGCAGGAAGCCAAATCT
pcDNA 3.1 CHK1-L	F-CCGGAATTCATGGCAGTGCCCTTTGTGG R-CGGGGTACCGATGTGGCAGGAAGCCAAATCT
CHK1-L	F-GGTGCCTATGGAGAAGTTCAA Probe- CAATCTTCACTGCGACTGCTTCTTCAG R-TCTACGGCACGCTTCATATC
CHK1-S	F-GTGCAAACCCTGGGAGAA Probe-TGCCTATGTCTGGCTTCTCCATAGGC R-CTCTGAGCATCTGGTTCAGG
ACTIN	F-ACCTTCTACAATGAGCTGCG Probe- ATCTGGGTCATCTTCTCGCGGTTG R-CCTGGATAGCAACGTACATGG
SRPK1	F- CGGTTGCTGAAGTCAGTTCG R- ACTTGAGCAGATGATGCCCC
ACTIN	F- CTACAATGAGCTGCGTGTGGC R- CAGGTCCAGACGCAGGATGGC

**Table 3:** Univariate and multivariate analysis of factors associated with overall survival of HCC patients.

Variable	Cox			
	Univariate analysis		Multivariate analysis	
	HR(95%CI)	p value	HR(95%CI)	p value
Age	0.991(0.968-1.016)	0.483		
Gender	1.542(0.651-3.652)	0.325		
Tumor size	1.109(0.981-1.254)	0.097		
Microvascular invasion	3.954(2.010-7.779)	0.000	3.929(1.959-7.879)	0.000
Differentiation	1.559(0.820-2.961)	0.175		
Satellite Nodules	2.941(1.362-6.351)	0.006		
Envelope invasion	1.924(0.920-4.022)	0.082		
Cirrhosis	1.068(0.451-2.525)	0.882		
AFP	1.052(0.959-1.153)	0.284		
CHK1-S in tumor tissue	0.857(0.763-0.963)	0.010		
CHK1-L in tumor tissue	0.894(0.765-1.044)	0.157		
CHK1-S in adjacent normal tissue	0.918(0.842-1.001)	0.053		
CHK1-L in adjacent normal tissue	0.923(0.816-1.044)	0.200		
Ratio of CHK1-S/L in tumor tissue	0.694(0.540-0.890)	0.004	0.685(0.524-0.897)	0.006
Ratio of CHK1-S/L in adjacent normal tissue	0.786(0.630-0.982)	0.034		
