# Supplementary material for: Clinical and functional significance of CHK1-S, an alternatively spliced isoform of the CHK1 gene, in hepatocellular carcinoma
Source: J Cancer. 2020 Jan 17;11(7):1792–9. doi: 10.7150/jca.39443 (PMC7052871; doi:10.7150/jca.39443)
Supplement: Supplementary file 1 — Supplementary figures and tables. [file jcav11p1792s1.pdf]

Supplementary table 1. List of differentially-expressed genes associated with alternative splicing in HCC.

Gene Symbol	Fold Change (linear) (tumor vs. normal)	ANOVA p-value (tumor vs. normal)	Description
HNRNPAB	2.18	0.001738	heterogeneous nuclear ribonucleoprotein A/B
MBNL2	-2.19	0.02638	muscleblind-like splicing regulator 2; muscleblind-like 2 (Drosophila)
RBM34	2.24	0.000116	RNA binding motif protein 34
RBMS3	-3.06	0.001742	RNA binding motif, single stranded interacting protein 3; RNA binding motif, single stranded interacting protein
RRM2	2.2	0.00235	ribonucleotide reductase M2; ribonucleotide reductase M2 polypeptide
SRPK1	2.04	0.003844	SRSF protein kinase 1
